# Screening and management of major endocrinopathies during pregnancy: an update

**DOI:** 10.1007/s12020-022-03237-y

**Published:** 2022-11-03

**Authors:** Stavroula A. Paschou, Evanthia Bletsa, Maria Papazisi, Nikoletta Mili, Fotini Kanouta, Georgia N. Kassi, Theodora Psaltopoulou, Dimitrios G. Goulis, Irene Lambrinoudaki

**Affiliations:** 1 Endocrine Unit and Diabetes Center, Department of Clinical Therapeutics, Alexandra Hospital, School of Medicine, National and Kapodistrian University of Athens, Athens, Greece; 2 Third Department of Cardiology, Sotiria Hospital, School of Medicine, National and Kapodistrian University of Athens, Athens, Greece; 3 School of Medicine, National and Kapodistrian University of Athens, Athens, Greece; 4 Second Department of Obstetrics and Gynecology, Aretaieion University Hospital, School of Medicine, National and Kapodistrian University of Athens, Athens, Greece; 5 Department of Endocrinology, Alexandra Hospital, Athens, Greece; 6 1st Department of Obstetrics and Gynecology, School of Medicine, Aristotle University of Thessaloniki, Thessaloniki, Greece

**Keywords:** Pregnancy, Gestational diabetes mellitus, Thyroid function, Calcium, Vitamin D

## Abstract

Endocrinopathies during pregnancy constitute a challenging issue, being prevalent and requiring appropriate management to avoid maternal and fetal complications. This review aims to summarize and present major endocrine problems during pregnancy, the appropriate screening, maternal monitoring and management, fetal monitoring, and follow-up. Glucose metabolism, thyroid function, as well as calcium and vitamin D metabolism are the main endocrine domains that should be screened and monitored during pregnancy. Gestational diabetes mellitus (GDM) is the most prevalent endocrine disease during pregnancy, followed by thyroid disorders. Specific recommendations are provided for the optimal clinical care of pregnant women and their offspring for GDM, thyroid disorders, and calcium and vitamin D disorders.

## Introduction

Endocrine disorders and their management during pregnancy pose therapeutic challenges for endocrinologists and obstetricians due to their potentially harmful maternal and fetal health effects. Gestation results in physiological endocrine changes, attributed to the placental function and the fetal-maternal interaction. Fetal endocrine gland development and hormone secretion are completed during the second trimester of pregnancy. Before this hallmark, fetal hormonal needs depend solely on maternal contribution, which remains substantial during pregnancy. Endocrine parameters that must be examined during pregnancy are glucose metabolism, thyroid function, and calcium and vitamin D metabolism. The respective endocrine disorders can be categorized into pre-existing and gestation-induced. The diagnosis is often demanding due to the overlap of symptoms between normal pregnancy and endocrine disease. This review summarizes the literature and guidelines regarding screening and managing of major endocrinopathies during pregnancy, providing a comprehensive, updated and clinically-oriented guide for endocrinologists and obstetricians.

## Study design and methods

To identify publications on endocrine disorders during pregnancy, a literature search was conducted in PubMed up to September 2022. Attention was paid to relevant guidelines. Information regarding screening and management of major endocrinopathies during pregnancy was collected, analyzed and qualitatively re-synthesized.

## Glucose metabolism disorders

### Pathophysiology

Hyperglycemia during pregnancy is classified into overt or pre-gestational diabetes mellitus (PGDM) and gestational diabetes mellitus (GDM). GDM is defined as glucose intolerance of variable degree with onset during pregnancy, usually during the second trimester, that was not PGDM. Pregnancy is characterized by a high risk of developing glucose metabolism disorders, considering that insulin resistance increases from the 24th to 28th gestational week. GDM is prevalent, complicating 7–8% of pregnancies [1].

Fetal glucose needs are met through maternal glucose transport via the placenta due to the concentration gradient between them. As the pregnancy progresses, a rise in fetal glucose requirements results in increased maternal hepatic glucose production and transport [2]. Simultaneously, the pancreatic β-cells are increased in mass to promote insulin production and prevent excessive glucose delivery to the fetus [3]. Failure of this process leads to hyperglycemia, usually between the 24th and 28th gestational weeks.

GDM has been strongly linked with reversible and non-reversible adverse effects for the mother and the fetus. On the one hand, pregnancies complicated with GDM are correlated with early complications, such as preeclampsia, spontaneous miscarriage, preterm birth, dystocia, and increased cesarian section rates. Later, women with GDM are susceptible to developing cardiovascular disease, type 2 diabetes mellitus (T2DM) and GDM in a subsequent pregnancy. On the other hand, fetal complications may be apparent early, such as macrosomia, birth trauma, shoulder dystocia, hypoglycemia, hyperbilirubinemia, preterm birth, and stillbirth. Offspring have a higher prevalence of obesity, metabolic syndrome and T2DM [4].

### Screening for gestational diabetes mellitus

Identifying women at high risk for developing GDM is of clinical relevance. The main risk factors for developing GDM are advanced maternal age, obesity, abnormal weight gain in pregnancy, first-degree relative with T2DM, a history of GDM, macrocosmia (newborn > 4 kg), perinatal complications, and polycystic ovary syndrome [5]. Although screening women with low GDM risk may not be cost-effective, these women represent a small percentage; thus, identifying and excluding them from screening may add unnecessary complexity to the screening process [6]. Therefore, the US Preventive Services Task Force recommends universal GDM screening starting from the 24th gestational week [7].

Early glucose screening must be performed on all pregnant women during the first antenatal visit by measuring fasting glucose in venous blood to identify GDM and pre-existing T2DM. Under normal conditions, fasting glucose values do not exceed 92 mg/dl; if they are between 92 and 125 mg/dl, a diagnosis of GDM is made. If they exceed 125 mg/dl (a confirmatory test is needed the following day), overt T2DM is diagnosed [2]. In pregnant women with normal fasting glucose concentrations at first antenatal screening, an oral glucose tolerance test (OGTT) is performed between the 24th–28th gestational week [5, 8].

GDM screening in asymptomatic pregnancies consists of a 2-step or 1-step approach at the 24th–28th gestational week. According to the 2-step approach, widely used in the United States, venous glucose concentrations are measured 1 h after a 50 g oral glucose administration without regard to the time of day or previous meals. Glucose concentrations that meet or exceed the institutional screening threshold imply women at high risk of GDM, who demand further testing. The second step is a 100 g, 3 h OGTT: 100 g of glucose are given in the morning after an 8 h overnight fasting. The diagnostic threshold of the first step varies from 130 to 140 mg/dl. The lower threshold provides higher sensitivity but results in more false-positive results compared with the 140 mg/dl threshold; therefore, it should be considered in populations with higher GDM prevalence. Different glucose thresholds have been proposed for the second step [9, 10]: Carpenter and Coustan or National Diabetes Data Group sets can be applied.

According to the 1-step approach, all pregnant women undergo a 75 g 2 h OGTT. A diet rich in carbohydrates (minimum of 180 g/day) should have been followed for three days, while OGTT should be conducted after 10 h fasting. Blood samples are collected in the fasting state, and 60 and 120 min following the glucose load. The diagnostic threshold values are 92, 180, and 153 mg/dl, respectively (Table 1). GDM is confirmed when at least one of these values is abnormal [8]. There is no consensus among national and international organizations regarding the optimal diagnostic approach; the choice is usually based on local clinical practices. Nevertheless, the 1-step approach is usually preferred and applied, since it is more cost-effective and simplifies the screening process [11]. Although glycosylated hemoglobin (HbA_1c_) reflects the average blood glucose concentrations, it is not an accurate index during pregnancy, and has no strong correlation with the risk of complications [12].

**Table 1:** Diagnosis of pre-existing and gestational diabetes mellitus

Diagnosis of pre-existing diabetes						
Glycemic index				Threshold		
Fasting plasma glucose				≥126 mg/dl		
Random plasma glucose				≥200 mg/dl		
HbA_1c_				≥6.5%		
*Diagnosis of gestational diabetes mellitus*						
*2-step approach*				*1-step approach*		
*Glycemic index*		*Threshold* (mg/dl)		*Glycemic index*		*Threshold* (mg/dl)
50 g OGCT	1 h	≥130–140		75 g OGTT ^§^		
		NDDG	Carpenter-Coustan			
100 g OGTT ^*^	Fasting	≥105	≥95		Fasting	≥92
	1 h	≥190	≥180		1 h	≥180
					2 h	≥153
	2 h	≥165	≥155			
	3 h	≥145	≥140			

*OGCT* oral glucose challenge test, *OGTT* oral glucose tolerance test, *NDDG* National Diabetes Data Group

^*^Two or more values for diagnosis

^§^One value for diagnosis

### Management of gestational diabetes mellitus

Appropriate management of hyperglycemia during pregnancy reduces maternal and fetal complications. According to American Diabetes Association (ADA) guidelines, therapeutic options for GDM are dietary changes, physical exercise, and insulin therapy [13]. Notably, metformin is not recommended as a first-line agent by ADA. On the contrary, the National Institute for Health and Care Excellence (NICE) guidelines recommend using metformin as a first-line agent in women whose diabetes is not adequately controlled by diet, before the institution of insulin therapy [14].

The first therapeutic step is conservative, based on mild exercise, such as walking for at least 30 min/day, unless contraindicated, together with a low-glycemic diet, consisting of three main meals and three snacks. The total caloric intake starts from 1800 kcal/day, consisting of 35–45% carbohydrates, 20–25% proteins, and 30–40% fat. Women diagnosed with GDM are advised to perform self-measurements of fasting (target < 95 mg/dl) and postprandial (target < 140 mg/dl) capillary glucose concentrations. Postprandial blood glucose should be monitored 1 h after starting a meal [5].

Findings from fetal ultrasound regarding fetal estimated weight, abdominal circumference, and amniotic fluid contribute to the decision-making. In more detail, insulin therapy initiation is recommended if 10–20% or more of glucose concentrations are abnormal after two weeks of appropriate diet and exercise. Ultrasound findings suggesting fetal macrosomia or increased amniotic fluid indicate the need for insulin initiation. In women with elevated fasting and postprandial glucose concentrations, the recommended starting total dosage is 0.7–1.0 units/kg/day, administered by multiple injections combining long- or intermediate-acting and short-acting insulin. When hyperglycemia occurs at a specific time, insulin administration should target to correct those values. NPH, detemir, regular, lispro, aspart and insulin degludec (recently approved) are safe to use during pregnancy [8, 15]. Post-partum insulin is discontinued in case of GDM, and women are encouraged to breastfeed. Women with a GDM history have a nearly 10-fold higher risk of T2DM compared with those with a normoglycemic pregnancy [16]. Therefore, it is recommended to perform a 75 g OGTT three months post-delivery. If the results are normal, the OGTT should be repeated in one year and, after that, every three years [8].

Oral and non-insulin injectable anti-diabetic agents are generally contradicted as they cross the placenta. Metformin crosses the placenta to the fetus [17], and its concentrations are similar to or higher than those in the pregnant woman; as a result, some safety issues remain [18]. In more detail, metformin may act as an endocrine disruptor, affecting the expression of gonad-related genes [19]. Metformin may also inhibit mitochondrial activity and result in nutrient restriction, negatively affecting the function, growth, or differentiation of fetal or placental tissues [20]. Mild nutrient prenatal restriction accompanied by nutrient excess in childhood may predispose an increased risk of obesity. Notably, metformin exposure during pregnancy results in higher BMI (body mass index), waist-to-height ratio, and waist circumference, as well as borderline increased fat mass in the offspring compared with insulin exposure [21–23]. Furthermore, metformin offers inadequate glycemic control for the treatment of GDM compared with insulin [24, 25].

ADA guidelines do not recommend metformin as a first-line agent during pregnancy [13], as its long-term safety for the offspring is unknown. The latter remains to be evaluated by studies in adults exposed to metformin in utero [19]. Metformin should be discontinued by the end of the first trimester, when used to treat polycystic ovary syndrome and induce ovulation [19], since it does not reduce the risk of spontaneous abortion or GDM in high-risk women with obesity, pre-existing insulin resistance or polycystic ovary syndrome [26, 27].

Nevertheless, metformin can be used to treat GDM under specific circumstances. As it crosses the placenta, metformin improves glucose levels and insulin resistance in the fetus, leading to a reduced risk of large-for-gestational-age (LGA) infants, neonatal hypoglycemia, respiratory distress syndrome and pregnancy loss [28]. The use of metformin during pregnancy has been associated with a reduced risk of neonatal hypoglycemia and maternal weight gain [29–31]. Metformin reduces maternal and neonatal hypoglycemia, and admissions in newborn intensive care unit compared with insulin [32]. Moreover, a recent systematic review and a meta-analysis indicated that metformin during pregnancy is strongly correlated with a lower maternal weight (mean difference −1.14 kg, 95% CI −2.22 to −0.06), though shorter gestational age at delivery (mean difference −0.16 weeks, 95% CI −0.30 to −0.02) compared with insulin [29]. Furthermore, a randomized, placebo-controlled trial demonstrated that metformin as add-on therapy in insulin-resistant pregnant women led to improved glycemic control and a significant reduction in gestational gain weight, fewer cesarean births and lower insulin requirements compared with placebo [33]. Additionally, infants of mothers taking metformin weighed less, were less likely to be extremely LGA, to weigh 4000 g or more at birth, and displayed decreased adipose and fat mass. On the other hand, metformin can decrease the risk of pre-eclampsia. According to a systematic review and meta-analysis of 8 randomized controlled trials, metformin use during pregnancy is strongly associated with a reduced risk of preeclampsia (RR 0.68, 95% CI 0.48–0.95; *p* = 0.02; I^2^ = 0%] compared with insulin [34].

According to NICE guidelines, women with diabetes may be advised to use metformin as an adjunct or alternative to insulin during pregnancy, when the likely benefits from improved blood glucose control outweigh the potential for harm [14]. Likewise, according to the ADA recommendations, metformin can be offered as an alternative option in women with GDM, who may not be able to use insulin safely and effectively, due to cost, language barriers and unwillingness for an injectable therapy, after discussion of the known risks and proper counseling [13]. Nevertheless, metformin should be avoided in women with hypertension or preeclampsia or at high risk for intrauterine growth restriction (IUGR) due to the potential for growth restriction or acidosis in the background of placental insufficiency [35]. Understanding the risks and benefits of metformin on maternal and fetal health will help clinicians to advise and manage women with GDM properly.

**Fig. 1:**
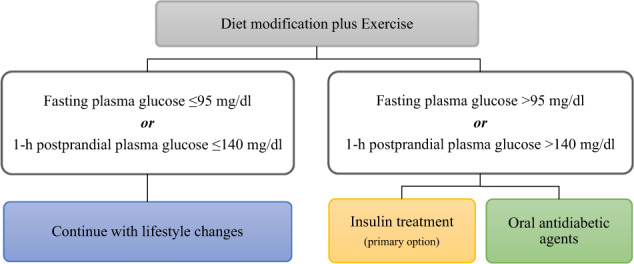
Management of gestational diabetes mellitus

Women with PGDM should replace oral medication with insulin, while type 1 diabetes mellitus (T1DM) requires initiation of insulin suitable for pregnancy or implementation of an insulin pump. As insulin resistance increases during the second trimester, the adaptation of insulin doses is important. General recommendations for women with PGDM are similar to those with GDM. However, glycemic targets are slightly different: ≤100 in the fasting state, ≤130 at 60 min postprandially, and ≤140 mg/dl at 120 min) [1, 36]. Contaminant medication, such as statins, should be interrupted during gestation, even if dyslipidemia is present, while only safe for pregnancy anti-hypertensive agents should be used to avoid adverse fetal outcomes (Fig. 1).

## Thyroid disorders

### Pathophysiology

Thyroid hormones are important in early embryogenesis for fetal brain development, embryo growth, and tissue differentiation. The fetal thyroid gland begins to form during the 4th gestational week, but it does not function until the second trimester. Maternal thyroxine still accounts for 30% of fetal serum thyroxine at term [37, 38].

During pregnancy, physiological hormonal changes and increased metabolic needs result in alterations in thyroid physiology [increased estrogen and human chorionic gonadotropin (hCG) concentrations, placenta development, increase in plasma volume and renal clearance]. Increased estrogens lead to an elevated hepatic production of serum thyroxine-binding globulin (TBG), which reaches a plateau during the 20th week. As a result, gradually higher total triiodothyronine (T_3_) and thyroxine (T_4_) serum concentrations are observed up to the 20th week (1.5 of threshold) [39]. Moreover, hCG, which has structural similarities to thyroid-stimulating hormone (TSH), stimulates TSH receptors, leading to elevated thyroid hormone production and reduced serum TSH concentrations via negative feedback. Therefore, TSH is completely suppressed in 20% of pregnant women, while another 2% may develop temporary hyperthyroxinemia. Furthermore, the development of the placenta, which results in the degradation of T_3_ and T_4_, the increase in plasma volume, and the increase in renal clearance, leads to elevated T_4_ production (50% approximately) and a similar increase in total daily iodine requirements. Thyroid hormones are converted into active forms (T_4_ to T_3_) via deiodinase, whose concentration and activity are upregulated by the placenta [37]. The thyroid gland is enlarged, especially if there is iodine deficiency [38]. These pathophysiological changes may lead to gestation-induced thyroid disorders or deregulation of pre-existing thyroid dysfunction.

### Screening for thyroid function

There is no consensus on whether universal thyroid function screening during pregnancy should be performed. American Thyroid Association (ATA) and American College of Obstetricians and Gynecologists (ACOG) suggest that screening should be offered in high-risk subgroups, such as those with a history of thyroid dysfunction, infertility, head/neck radiation, obesity or clinical suspicion of thyroid disease [37, 40].

TSH is the gold standard test to screen for thyroid function in pregnant women. Due to the physiologic changes in thyroid hormone concentrations during pregnancy, a trimester-specific reference range is necessary. TSH concentrations may be misleading during the first trimester, but they are accurate in the following two. Trimester-specific TSH thresholds are: first trimester 0.1–2.5 mU/l, second trimester 0.2–3.0 mU/l [Endocrine Society, ATA, European Thyroid Association (ETA)] and third trimester 0.3–3.0 mU/l (Endocrine Society, ATA) or 0.2–3.5 mU/l (ETA) (Table 2). In case of abnormal TSH concentrations, thyroid hormones should be measured to diagnose overt thyroid dysfunction. Free thyroid hormones are preferred to total, since the latter depend largely on the TBG concentration [41]. However, when investigating hyperthyroidism, total T_3_ is preferred over fT_3_ [42]_._ In euthyroid women, routine testing for autoantibodies to thyroid peroxidase (anti-TPO) and thyroglobulin (anti-Tg) is not recommended.

**Table 2:** TSH threshold values in pregnancy

	Endocrine Society	European Thyroid Association
	American Thyroid Association	
Trimester	TSH (mU/l)	
First	0.1–2.5	0.1–2.5
Second	0.2–3.0	0.2–3.0
Third	0.3–3.0	0.2–3.5

### Hypothyroidism

Primary overt maternal hypothyroidism is defined as the presence of an elevated TSH and a decreased serum fT_4_ concentration during pregnancy. Subclinical hypothyroidism is defined by fT_4_ concentrations within the reference range. Inversely, isolated hypothyroxinemia is defined as low fT_4_ concentrations accompanied by normal TSH concentrations [37].

Subclinical hypothyroidism (TSH > 4 mU/l) has a prevalence of 2–3% among pregnant women, while 0.3–0.5% present with overt hypothyroidism [38]. Up to 15% of pregnant women may develop TSH concentrations above 2.5 mU/l. The most common causes of overt hypothyroidism are iodine deficiency, worldwide, and chronic autoimmune thyroiditis (Hashimoto thyroiditis) in the developed world. Most women remain asymptomatic; however, some present with fatigue, constipation, weight gain, and cold intolerance [37]. Untreated overt hypothyroidism is a risk factor for miscarriage, preeclampsia, GDM, placental abruption and preterm labor, whereas subclinical hypothyroidism has been associated with fewer adverse pregnancy outcomes [38].

### Management of hypothyroidism

Replacement therapy with levothyroxine (LT_4_) should be given to pregnant women with overt hypothyroidism to reduce the risk of maternal and fetal complications. If TSH does not exceed threshold values, LT_4_ replacement is generally contradicted in pregnant women with positive anti-TPO or anti-Tg. Full replacement therapy is achieved with LT_4_ at 1.1 μg/kg/day. LT_4_ dosage should be adapted to reach and maintain serum trimester-specific TSH concentrations [40]. Thyroid function tests should be repeated every 4–6 weeks. If hypothyroidism has been diagnosed before pregnancy, adjusting the preconception daily LT_4_ dose is suggested: most women require a 25–30% increase in dosage after the 5th–6th gestational week [41]. Postpartum, some women decrease the T_4_ dosage to the preconception level, while 75% may quit LT_4_ [41].

LT_4_ treatment for subclinical hypothyroidism among pregnant women remains controversial. LT_4_ therapy is associated with a reduced risk of pregnancy loss among pregnant women with subclinical hypothyroidism [43, 44]. However, subgroup analysis indicated that the benefit of treatment on pregnancy was only observed in women with a TSH above 4.0 mU/l at baseline [44]. Similarly, LT_4_ treatment for pregnant women with subclinical hypothyroidism (TSH > 4.0 mU/l) at the first prenatal visit with or without TPO-antibody positivity leads to a significant reduction in preterm birth [45, 46]. A meta-analysis indicated a decreased risk of pregnancy loss (OR 0.78, 95% CI 0.66–0.94) and increased risk of preterm labor (OR 1.82, 95% CI 1.14–2.91) in women with subclinical hypothyroidism treated with LT_4_, leading to debates regarding the treatment of subclinical hypothyroidism in pregnancy [47]. The heterogeneity of results is attributed to differences in LT_4_ doses, the timing of treatment initiation, and definitions of subclinical hypothyroidism in each study. As a result, the Endocrine Society (US) recommends treatment of subclinical hypothyroidism irrespective of anti-TPO status [36]. On the other hand, ATA guidelines suggest that LT_4_ therapy may be considered for anti-TPO positive women with TSH concentrations >2.5 mU/l and below the upper limit of the pregnancy-specific reference range or for anti-TPO negative women with TSH > 10 mU/l [48]. However, this recommendation remains weak.

### Hyperthyroidism

Subclinical hyperthyroidism is defined by decreased serum TSH concentration and normal fT_4_ concentrations. With a 1–2% prevalence, subclinical hyperthyroidism has not been associated with adverse pregnancy outcomes [49, 50]. On the other hand, overt hyperthyroidism, defined as decreased TSH and increased fT_4_ concentrations, is rare during pregnancy (0.2–0.7%); Grave’s disease is the cause of 95% of these cases [49]. Considering the changes in thyroid function during gestation, low TSH and high fT_4_ and fT_3_ concentrations are well-tolerated, especially during the first trimester. Hyperthyroidism symptoms include palpitations, diaphoresis, heat intolerance, and weight loss [48]. Untreated overt hyperthyroidism is a risk factor for preeclampsia, maternal heart failure and thyroid storm, fetal thyrotoxicosis, preterm birth, low birth weight, miscarriage, and stillbirth [51, 52].

A low serum TSH concentration during gestation could be the outcome of physiological gestational hormonal changes or indicate hyperthyroidism. Suggestive of the latter is the clinical evidence of autoimmunity or thyroid receptor antibodies [TRAb or thyroid-stimulating immunoglobulins (TSI)] or a goiter. TRAb should be measured between the 24th and 28th gestational week in pregnant women with a neonate with hyperthyroidism, a history of Graves’ disease or current Graves’ disease, thyroidectomy before pregnancy, treatment with ^131^I due to hyperthyroidism, or fetal tachycardia accompanied by goiter IUGR [39]. In patients with Graves’ disease, TRAb and TSI may indicate those at higher risk for fetal and maternal complications [40]. As a result, a high index of clinical suspicion is necessary for early detection. Thyroid scan with ^99m^Tc is contradicted during pregnancy. When thyrotoxicosis concerns T_3_, total T_3_ concentrations should be monitored as an index of thyroid function [48].

### Management of hyperthyroidism

Treatment of hyperthyroidism due to Graves’ disease or toxic thyroid nodules involves anti-thyroid drugs (ATDs) and aims to regulate the symptoms and maintain maternal fT_4_ concentrations in the upper limit of normal. Thionamide ATDs [methimazole (MMI), carbimazole (CBZ), propylthiouracil (PTU)] can be used during pregnancy. Treatment with the lowest effective dose aims to maintain fT_4_ concentrations within the high-normal range, regardless of TSH concentrations, to avoid fetal hypothyroidism. In case of pre-existing or newly diagnosed hyperthyroidism demanding therapy during pregnancy, PTU and MMI are recommended during the first and second/third trimester, respectively.

MMI and PTU may have adverse effects on the gravida [41]. MMI has been associated with potential teratogenic effects, such as aplasia cutis and other congenital malformations, since it crosses the placenta [53]. As a result, MMI should be avoided during the first trimester. A woman on ATDs for Grave’s disease, currently euthyroid, could attempt to discontinue therapy and monitor the thyroid function closely during the first trimester. Especially during the 6th–10th gestational weeks, when ATDs have the greatest teratogenic effect, drug suspension could be recommended. However, if this is not possible, PTU is considered the first-line agent during the first trimester, whereas MMI is preferred in the second and third trimesters. If a patient has a severe adverse reaction or is non-adherent to ATDs and has uncontrolled hyperthyroidism, thyroidectomy is recommended, optimally in the second trimester [41, 48]. In the rare case of fetal hyperthyroidism, MMI, PTU, and CBZ effectively cross the placenta; therefore, ATDs for maternal hyperthyroidism also modulate fetal thyroid function [48]. Notably, the effect of ATDs is more potent in the fetus than in the mother [54]. To avoid deleterious fetal complications, the aim is to maintain maternal fT_4_ concentrations at or just above the pregnancy-specific upper normal limit, using the lowest effective dose of ATDs [54].

### Postpartum thyroiditis

Of pregnant women with positive anti-TPOs or anti-TGs, 4–10% will develop autoimmune post-partum thyroiditis within the first year after delivery [41]. It is characterized by temporary hyperthyroidism due to the destruction of thyroid parenchyma, followed by hypothyroidism, presenting mild, non-specific symptoms. The diagnosis is based on new-onset TSH and fT_4_ concentrations outside the normal range. This state should be distinguished from Graves’ disease, which demands therapy with ATDs [41]. Most commonly, transient postpartum thyroiditis will resolve spontaneously; after transient hypothyroidism, euthyroidism will be restored. However, in one-third of affected women, thyroiditis will persist and evolve into permanent, overt hypothyroidism, which may demand LT_4_ replacement.

## Vitamin D and calcium metabolism disorders

### Pathophysiology

Vitamin D, produced in the skin by direct exposure to ultraviolet B radiation or received through fortified food intake, undergoes hydroxylation to form the active 125(OH)_2_D_,_ regulating calcium homeostasis and bone health. The active vitamin D promotes calcium and phosphorus intestinal absorption and deposition to the bones, and reduces their renal clearance [55]. Increased calcium demands during gestation are met through increased absorption and mobilization from the skeleton, while renal absorption remains unchanged [56]. Calcium metabolism during pregnancy aims to satisfy the needs of the developing fetus, such as the mineralization of the skeleton, through the active transfer of calcium across the placenta [56]. During the third trimester, 80% of calcium transfer occurs via the placenta. Biomarkers of bone absorption (NTx, CTx) and production (P1NP, bALP) are increased during the first and third trimester of pregnancy, respectively; an increase in 1,25(OH)_2_D is also observed. Parathyroid hormone-related protein (PTHrP), secreted by the mammary gland, placenta, and myometrium, facilitates calcium transfer via the placenta. PTHrP activates the PTH/PTHrP receptor and induces 1α-hydroxylation to produce the active form of vitamin D.

Vitamin D deficiency in pregnancy has been associated with an increased risk of adverse pregnancy outcomes, such as preeclampsia, GDM, low birth weight and preterm birth [57]. Vitamin D deficiency is very prevalent among pregnant women, especially in populations with risk factors such as darker skin, limited sun exposure due to residing in cold climates and northern regions or wearing protective garments, and vegetarians [58, 59]. Thus, early detection of calcium metabolism disorders is important to prevent serious maternal and fetal complications.

### Hypovitaminosis D

25(OH)D serum concentrations can be used as an index of vitamin D status in pregnant women. There is no consensus on the threshold that indicates deficiency. Recent evidence suggests that 25(OH)D concentrations should be maintained above 30 ng/ml to avoid pregnancy outcomes [60]. Vitamin D deficiency in pregnant and lactating women should be managed with up to 4000 IU of vitamin D_3_ daily [61, 62]. Consumption of low-fat dairy foods for sufficient calcium intake is advised. Calcium supplementation in doses of 500–1000 mg daily is recommended, especially during the second and third trimesters, considering the increased need for calcium during this period. High-dose calcium supplementation (≥1 g/day) is recommended for women at high risk of gestational hypertension in areas with low calcium intake [63].

Increased calcium needs occur during lactation, as calcium is transferred via breast milk. Increased bone and renal reabsorption but not elevated intestinal adsorption during pregnancy are the main mechanisms for maintaining calcium homeostasis during lactation. The strongest regulators are increased PTHrP concentrations secreted by the mammary gland and reduced estradiol concentrations due to hyperprolactinemia. Even one year after terminating breastfeeding may be required to achieve a positive balance in calcium metabolism. Subsequently, calcium supplementation in doses of 500–1000 mg daily is recommended during lactation; 25(OH)D concentrations should be maintained above 30 ng/ml.

### Primary hyperparathyroidism

Parathyroid gland disorders are rarely seen during pregnancy and lactation. Primary hyperparathyroidism (PHPT) resulting in clinical hypercalcemia is mainly due to single adenoma, less commonly to hyperplasia and rarely to malignancy. PHPT has been associated with maternal and fetal complications, such as hypercalcemic crisis, preeclampsia, hypertension, IUGR, low birth weight, preterm birth, stillbirth, and increased perinatal death rates (up to 30%). Most affected pregnant women present with nausea, vomiting, weight loss, anorexia, weakness, and fatigue. Other symptoms include headaches, lethargy, agitation, and confusion. Nephrolithiasis, bone disease, acute pancreatitis and new-onset hypertension can also be present; less than 25% of patients are asymptomatic [64].

Considering the normal decrease in calcium concentrations during pregnancy, diagnosis of hypercalcemia and PHPT is usually delayed and based on persistently increased serum calcium (>9.5 mg/dl) and increased PTH concentrations. Increased 24 h urinary calcium excretion can be an indicator for screening for PHPT. Close monitoring, hydration and avoidance of medications that could elevate calcium constitute the recommended management for asymptomatic pregnant women with serum calcium concentrations <1 mg above the normal range. In symptomatic patients with complications and persistent hypercalcemia, surgical removal is recommended during the second trimester [56], while surgical options should be offered in asymptomatic women post-partum, before the next pregnancy.

### Hypoparathyroidism

Hypoparathyroidism is caused by damage or removal of parathyroid glands after thyroid surgery; other rare causes are idiopathic and autoimmunity. The resulting hypocalcemia leads to a further drop in maternal calcium concentrations during pregnancy. Thus, it can be detrimental to fetal skeletal development, leading to intrauterine death. Maternal manifestations of hypocalcemia, such as muscle tension, paresthesia, and tetany, have been reported during pregnancy [64, 65].

Diagnosis of hypoparathyroidism is based on persistently low PTH and calcium accompanied by high serum phosphate concentrations. Severe hypocalcemia symptoms include paresthesia, carpopedal spasm, stridor, dyspnea, and seizures. Chvostek and Trousseau signs may be present [64]. Treatment of hypoparathyroidism is based on calcium and vitamin D supplementation, aiming to achieve low-normal concentrations of serum ionized calcium concentration or calcium corrected for albumin [65]. The most used combination is high-dose calcium supplementation (1–2 g/day) with calcitriol (1–3 μg/day). Vitamin D (cholecalciferol) can also be used in a weekly dose of 50,000–150,000 IU [64].

### Osteoporosis

Osteoporosis is a rare clinical condition during pregnancy or lactation. It is observed among women with major risk factors or secondary osteoporosis. A rare type of osteoporosis associated with pregnancy should be mentioned. It is a temporary condition, usually located in the hip during the third trimester of pregnancy. The main symptoms are unilateral or bilateral hip pain, dystrophy, limited mobility, difficulty in walking and hip fracture. It is attributed to local malfunction factors, such as vascular congestion, nerve compression, and sympathetic system dysfunction; it is usually self-limited.

## Conclusions

Endocrine disorders are among the most prevalent pregnancy complications with possible adverse maternal and fetal outcomes. If diagnosed early in pregnancy, they can be treated timely, avoiding further adverse outcomes. To offer appropriate maternal care and prevent severe complications, global and national health organizations have proposed screening and management guidelines. Clinicians should be aware of possible glucose metabolism, thyroid, calcium and vitamin D disorders during pregnancy and their optimal management.
